# All-case Japanese post-marketing surveillance of the real-world safety and efficacy of rituximab treatment in patients with refractory nephrotic syndrome

**DOI:** 10.1007/s10157-021-02035-6

**Published:** 2021-04-01

**Authors:** Mana Kobayashi, Yutaro Kageyama, Takashi Ando, Junko Sakamoto, Shohji Kimura

**Affiliations:** Pharmacovigilance and Quality Assurance Department, Zenyaku Kogyo Co., Ltd, 6-15, Otsuka 5-Chome, Bunkyo-ku, Tokyo, 112-8650 Japan

**Keywords:** Frequent relapsing, Japan, Refractory nephrotic syndrome, Rituximab, Steroid-dependent

## Abstract

**Background:**

Rituximab is conditionally approved in Japan for use in patients with refractory nephrotic syndrome. To meet the conditions of approval, an all-case post-marketing surveillance study was conducted to confirm the real-world safety and efficacy of rituximab in patients of all ages with refractory nephrotic syndrome.

**Methods:**

All patients scheduled to receive rituximab treatment for refractory nephrotic syndrome were eligible to register (registration: August 29, 2014 through April 15, 2016); the planned observation period was 2 years from the initiation of rituximab treatment (intravenous infusion, 375 mg/m^2^ once weekly for four doses). The study was conducted at 227 hospitals throughout Japan. Adverse drug reactions (ADRs) were collected for safety outcomes. The efficacy outcomes were relapse-free period and the degree of growth in pediatric (< 15 years) patients.

**Results:**

In total, 997 (447 pediatric) patients were registered; 981 (445) were included in the safety analysis set; 852 (402) completed the 2-year observation period; and 810 (429) were included in the efficacy analysis set. Refractory nephrotic syndrome had developed in childhood for 85.0% of patients, and 54.6% were aged ≥15 years. ADRs were observed in 527 (53.7%) patients, treatment-related infection/infestation in 235 (24.0%) patients, and infusion reactions in 313 (31.9%) patients. The relapse-free period was 580 days (95% confidence interval, 511–664). There was a significant change in height standard deviation score (pediatric patients; mean change, 0.093; standard deviation, 0.637; *P* = 0.009).

**Conclusion:**

The safety and efficacy of rituximab treatment in patients with refractory nephrotic syndrome were confirmed in the real-world setting.

**Clinical trial registration:**

UMIN000014997.

**Supplementary Information:**

The online version contains supplementary material available at 10.1007/s10157-021-02035-6.

## Introduction

Nephrotic syndrome is a kidney disorder in which protein leaks from the blood (through the glomeruli) to the urine. It is characterized by excess protein excretion in the urine which results in hypoproteinemia, systemic edema, and dyslipidemia (elevated low-density lipoproteins) [1]. Cytokines produced by T cells are thought to act on the slit membrane to enhance protein permeability; however, the exact mechanisms underlying the development of nephrotic syndrome remain unknown [2].

In Japan, approximately 1300 new cases of nephrotic syndrome are reported annually to the Research Project for the Treatment of Chronic Pediatric Diseases, suggesting that 5 out of 100,000 children will develop nephrotic syndrome each year [3]. Childhood nephrotic syndrome occurs most often between the ages of 2 and 6 years [4]. Approximately 90% of these cases are idiopathic nephrotic syndromes [3], most of which are classified as minimal change disease (little change in the glomeruli when examined using light microscopy) and can be successfully treated with steroid therapy [2]. At least 20% of children with idiopathic nephrotic syndrome have frequent relapsing nephrotic syndrome (FRNS) or steroid-dependent nephrotic syndrome (SDNS); together these patients are referred to as having complicated FRNS/SDNS [5]. Patients with FRNS and SDNS are not able to discontinue or reduce steroid treatment without relapse and require concomitant immunosuppressant treatment [6]. The side effects of long-term steroid use are particularly problematic in growing children and long-term immunosuppressant use also comes with undesirable side effects [5, 7]. Additionally, in a small number of cases (1–3%), patients do not respond to treatment with immunosuppressants and progress to renal failure [5, 8]. Together, these issues highlight the need for a safer and more effective approach to treatment.

Rituximab is a human/murine, chimeric anti-CD20 monoclonal antibody that binds to CD20, which is expressed specifically on B cells, and exhibits cytotoxicity upon binding [9]. Two simultaneous trials were conducted in Japan to determine the safety and efficacy (RCRNS-01, randomized controlled trial; rituximab 31 patients, placebo 24 patients) (unpublished) and the pharmacokinetics (RCRNS-02, open label trial; 23 patients) (unpublished) of rituximab treatment in children with complicated FRNS/SDNS. A portion of the results from the RCRNS-01 study have been reported (rituximab 24 patients, placebo 24 patients) [10] and it was shown that the 50% relapse-free period in the rituximab group was significantly longer than in the placebo group (267 vs. 101 days; hazard ratio 0.27, 95% confidence interval [CI] 0.14–0.53; *P* < 0.0001) and that the rituximab safety profile in these patients was in line with the known safety profile of this drug.

Based on the results of these studies, rituximab was approved for use in these patients by the Ministry of Health, Labor and Welfare of Japan in August 2014. This approval came with the condition that an all-case post-marketing survey must be conducted until the appropriate amount of data were collected to confirm the safety and efficacy of rituximab in patients of all ages with refractory nephrotic syndrome (FRNS or SDNS), due to the small number of these patients who were included in the randomized controlled clinical trial. Therefore, this all-case post-marketing surveillance was conducted in Japan to confirm the real-world safety and efficacy of rituximab in patients of all ages with refractory nephrotic syndrome (FRNS or SDNS). All patients who had refractory nephrotic syndrome and were treated with rituximab in Japan during the study enrollment period were targeted for inclusion in this analysis.

## Methods

### Patients

All patients scheduled to use rituximab for the treatment of refractory nephrotic syndrome were eligible to register for this all-case post-marketing surveillance; no exclusion criteria were set. As this was a post-marketing surveillance study, it was not necessary to obtain approval from an ethical review board or patient informed consent, based on Japanese regulatory guidelines for Good Post-marketing Surveillance Practice [11].

### Study design

This was an all-case post-marketing surveillance study conducted in Japan to determine the real-world safety and efficacy of rituximab treatment in all patients with refractory nephrotic syndrome who were scheduled for treatment with rituximab (clinical trial registration: UMIN000014997). This survey was conducted at all hospitals in Japan (*N* = 227) where patients with physician-diagnosed refractory nephrotic syndrome were treated with rituximab. The target number of pediatric patients was 300; pediatric patients were defined as aged < 15 years at the time of the initial rituximab infusion. There was no target number set for adult patients (aged ≥ 15 years at the time of the initial rituximab infusion). The patient registration period was August 29, 2014 (the date of rituximab approval for use in patients with refractory nephrotic syndrome), through April 15, 2016. Patients were registered at least 3 days prior to initiating rituximab; treatment consisted of an intravenous infusion of 375 mg/m^2^ rituximab once weekly for four doses. The maximum single dose was 500 mg.

The surveillance study was conducted using a central registration system and the planned observation period was 2 years from initiation of rituximab treatment. Patient background, administration status of rituximab, concomitant drug status, clinical examination, adverse drug reactions (ADRs), the effectiveness of treatment, and patient height and weight at the end of the observation period were collected for analysis.

#### Primary endpoints

##### Safety

ADRs were collected for safety endpoints. The Medical Dictionary for Regulatory Activities (MedDRA; version 21.1) system organ class and preferred term were used to evaluate and categorize ADRs; grading was performed by the investigator according to the Common Terminology Criteria for Adverse Events (CTCAE; version 3.0).

The incidence of ADRs was examined according to patient population characteristics. The incidence of infusion reactions was examined according to the number of rituximab infusions, circumstances at the initial rituximab infusion, and patient background. Specific pathogens were determined for ADRs of infections and infestations; these ADRs were also examined according to risk factors for infectious disease.

##### Relapse-free period

The relapse-free period was considered to be the period from the date of rituximab administration to the date of nephrotic syndrome relapse. Relapse was defined as an early morning urine protein result of 3+ (using the test paper method [3]) for 3 consecutive days.

#### Secondary endpoint

##### Degree of growth

The degree of growth in pediatric patients (defined as < 15 years old at the initiation of administration) after rituximab administration was evaluated. Height standard deviation score (SDS) was calculated using the 2000 Infant Physical Development Report (Ministry of Health, Labor and Welfare, Japan) and the School Health Statistics 2000 (Ministry of Education, Culture, Sports, Science and Technology, Japan) report [12, 13].

### Statistical methods

There is limited experience with rituximab in the pediatric age group; therefore, a target number of 300 pediatric patients was determined as the number needed for 95% confidence that at least one ADR would occur in at least 1.0% of these patients. For adult patients (defined as aged ≥ 15 years at the time of the initial rituximab infusion), no target number was set as it was assumed that a sufficient number of these patients would be enrolled when pediatric enrollment reached 300.

The safety analysis set included all patients registered for the survey who did not meet the safety analysis exclusion criteria. The efficacy analysis set included all patients in the safety analysis set who did not meet the efficacy analysis exclusion criteria; both sets of exclusion criteria are listed in Online Resource 1.

Summary statistics were used to describe clinical characteristics and safety outcomes. The Chi-square test was used to perform risk analysis. In cases where the number of patients was too few for statistical analysis, those patients were excluded from risk analysis. A Kaplan–Meier curve was used to demonstrate the relapse-free period; the log-rank test was used for comparative analysis. Changes in height SDS were compared using a paired Student’s *t*-test. Two-tailed *P* values < 0.05 were considered statistically significant. Missing data were treated as unknown or were excluded. Statistical analyses were conducted using SAS version 9.2 or later (SAS Institute; Cary, NC, USA).

## Results

### Patient population

**Fig. 1:**
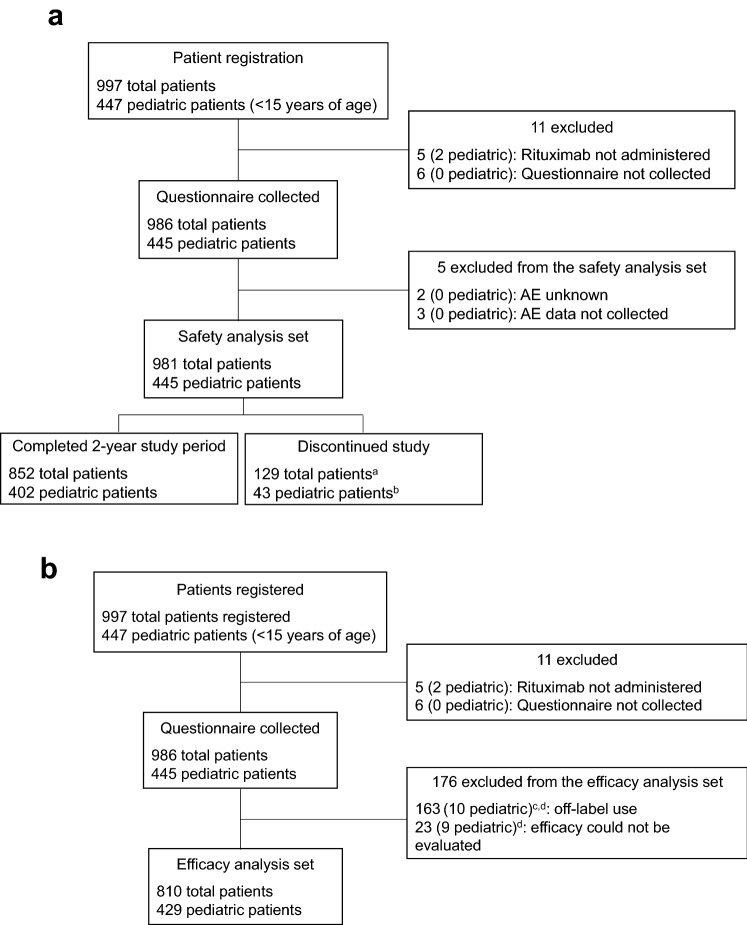
Flow diagram of the **a** safety analysis set, and **b** efficacy analysis set. *AE* adverse event. ^a^Non-recoverable AE, 14 patients; AE information incomplete, 15 patients; hospital transfer, 84 patients; death, two patients; other reason, 14 patients. ^b^AE information incomplete, 11 patients; hospital transfer, 28 patients; other, four patients. ^c^Off-label use for steroid-resistant nephrotic syndrome or adult-onset nephrotic syndrome. ^d^There were 10 patients (including three pediatric patients) who were counted in duplicate for both reasons (“off-label use” and “efficacy could not be evaluated”)

The investigation period was August 29, 2014 to October 15, 2018 (30 months after registration ended). In total, 997 patients were registered, 447 of whom were pediatric patients. Eleven registered patients (two pediatric) were excluded because either rituximab was not administered (five patients [two pediatric]) or because it was not possible to collect the questionnaire (six patients). Questionnaires were collected from the remaining 986 patients (445 pediatric). Flow charts for the safety and efficacy analysis sets are shown in Fig. 1a,b, respectively. Of the 981 patients (445 pediatric) included in the safety analysis set, 852 patients (402 pediatric) completed the 2-year observation period and 129 (43 pediatric) discontinued the study. The efficacy analysis set included 810 patients (429 pediatric); the main reason for exclusion was off-label use of rituximab. Two patients, both of whom were elderly, died during the investigation period. One of these patients had pneumonia that started 12 days after rituximab administration and died due to septicemia 4 months after rituximab administration. No information on the cause of death was available for the other patient who died because the death was reported to the hospital by the family of the patient and they did not disclose the cause.

The characteristics of the patient population are shown in Table 1. Of the patients included in the safety analysis set, 65.3% (641 of 981) were male; 10.0% (98 of 981) were < 7 years of age; 35.4% (347 of 981) were between 7 and < 15 years of age; and 54.6% (536 of 981) were ≥ 15 years of age. Most patients (85.0%, 834 of 981) developed refractory nephrotic syndrome in childhood (< 18 years of age). Rituximab was used to treat FRNS or SDNS in 59.9% (588 of 981) or 68.3% (670 of 981) of patients, respectively. The initial dose was 375 mg/m^2^ for 26.0% (255 of 981) of patients and 500 mg/person for 58.5% (574 of 981) of patients; 32.1% (315 of 981) of patients were treated with rituximab once and 67.9% (666 of 981) were treated twice or more. There were no patients with pulmonary dysfunction or severe bone marrow dysfunction. Additional information regarding the patient population, including the major reported complications and medical history, are reported in Online Resource 2.

**Table 1:** Patient population (safety analysis set, *N* = 981)

	Total *N* (%)	Pediatric *n*	Adult *n*
Sex			
Male	641 (65.3)	288	353
Female	340 (34.7)	157	183
Age (years)			
< 3 vs ≥ 3			
< 3	5 (0.5)	–	–
≥ 3	976 (99.5)	–	–
< 7 vs ≥ 7 to < 15 vs ≥ 15			
< 7	98 (10.0)	98	0
7 to < 15	347 (35.4)	347	0
≥ 15	536 (54.6)	0	536
≥ 65 vs < 65			
≥ 65	17 (1.7)	–	–
< 65	964 (98.3)	–	–
Nephrotic syndrome onset			
Childhood	834 (85.0)	445	389
Adulthood^a^	146 (14.9)	0	146
Congenital	1 (0.1)	0	1
Reason for rituximab use			
Frequent relapse^b^	588 (59.9)	276	312
Steroid dependence^b^	670 (68.3)	303	367
Steroid resistance^a^	24 (2.4)	10	14
Other^c^	2 (0.2)	0	2
Disease type			
Minimal change disease	321 (32.7)	127	194
Focal segmental glomerulosclerosis	53 (5.4)	16	37
Membranous nephropathy	8 (0.8)	0	8
Membranoproliferative glomerulonephritis	5 (0.5)	0	5
Other	8 (0.8)	3	5
Unknown	587 (59.8)	299	288
History of rituximab treatment			
No	735 (74.9)	350	385
Yes	244 (24.9)	93	151
Unknown	2 (0.2)	2	0
Cardiac dysfunction			
No	977 (99.6)	445	532
Yes	4 (0.4)	0	4
Treatment with an antihypertensive drug			
No	704 (71.8)	319	385
Yes	277 (28.2)	126	151
Drug hypersensitivity			
No	930 (94.8)	424	506
Yes	51 (5.2)	21	30

^a^Example of off-label use

^b^Includes 303 duplicate patients

^c^Intractable nephrosis, two patients with unknown details

#### Primary outcomes

##### Safety

ADRs were observed in 527 (53.7%) patients. Pediatric patients experienced ADRs of infections and infestations; respiratory, thoracic, and mediastinal disorders; and gastrointestinal disorders at rates ≥ 5% higher than adult patients. ADRs that were notably higher in the pediatric vs adult population were influenza and abdominal pain. Considering their time of onset, most respiratory and gastrointestinal disorders appeared to be infusion reactions.

Table 2 shows the occurrence of ADRs according to patient and treatment characteristics. Characteristics of the patient population that were significantly associated with the occurrence of ADRs were initial rituximab dose (375 mg/m^2^), number of rituximab administrations (higher), age at onset of refractory nephrotic syndrome (< 18 years), prior treatment with rituximab (no), and concomitant immunosuppressive drugs (yes). The incidence of serious ADRs according to patient and treatment characteristics is shown in Online Resource 3.

**Table 2:** Occurrence of adverse drug reaction according to patient and treatment characteristics (safety analysis set, *N* = 981)

	Total number of patients *N*	Patients experiencing ≥1 ADR *n* (%)	*P* value^a,b^	OR (95% CI)^b^
Total population	981	527 (53.7)		
Sex				
Male	641	332 (51.8)	0.097	1
Female	340	195 (57.4)		1.252 (0.960, 1.631)
Age (years)				
< 7	98	67 (68.4)	< 0.001	1
7 to < 15	347	221 (63.7)		0.812 (0.503, 1.310)
≥ 15	536	239 (44.6)		0.372 (0.235, 0.589)
No. rituximab treatments^c^				
1	315	152 (48.3)	0.256	1
2	232	131 (56.5)		1.391 (0.989, 1.956)
3	166	88 (53.0)		1.210 (0.830, 1.763)
4	187	107 (57.2)		1.434 (0.996, 2.065)
5	61	33 (54.1)		1.264 (0.729, 2.190)
6	15	12 (80.0)		–
7	3	3 (100.0)		–
8	1	0 (0.0)		–
9	1	1 (100.0)		–
Dose of rituximab (first use)				
375 mg/m^2^	255	164 (64.3)	< 0.001	1
500 mg/person	574	283 (49.3)		0.540 (0.398, 0.731)
450 mg/person	10	4 (40.0)		–
300 mg/person	19	14 (73.7)		–
200 mg/person	15	7 (46.7)		–
100 mg/person	43	13 (30.2)		–
Other	65	42 (64.6)		–
No. rituximab administrations				
1	183	85 (46.4)	0.017	1
2	167	84 (50.3)		1.167 (0.767, 1.776)
3	113	59 (52.2)		1.260 (0.788, 2.015)
4	287	152 (53.0)		1.298 (0.895, 1.882)
5	100	56 (56.0)		1.467 (0.899, 2.396)
6	53	35 (66.0)		2.242 (1.184, 4.245)
7	29	23 (79.3)		4.420 (1.719, 11.363)
8	35	22 (62.9)		1.951 (0.927, 4.108)
9	6	6 (100.0)		–
10	2	0 (0.0)		–
11	2	1 (50.0)		–
12	3	3 (100.0)		–
16	1	1 (100.0)		–
Initial infusion rate^d^ (mg/h)				
25	774	429 (55.4)	0.297	1
50	104	52 (50.0)		0.804 (0.534, 1.211)
< 25	63	28 (44.4)		
25–50	17	8 (47.1)		–
50–100	2	1 (50.0)		–
100	20	9 (45.0)		–
Unknown	1	0 (0.0)		–
Max infusion rate^d^ (mg/h)				
200	608	307 (50.5)	0.155	1
400	25	9 (36.0)		0.552 (0.240, 1.267)
< 100	98	55 (56.1)		–
100	194	130 (67.0)		–
100–200	41	20 (48.8)		–
200–400	13	5 (38.5)		–
Unknown	2	1 (50.0)		–
Age at refractory nephrotic syndrome onset (years)				
< 18	834	466 (55.9)	< 0.001	1
≥ 18	146	60 (41.1)		0.551 (0.386, 0.787)
Congenital	1	1 (100.0)		–
Immunosuppressant use^e^				
No	106	44 (41.5)	0.007	1
Yes	870	481 (55.3)		1.742 (1.158, 2.622)
Unknown	5	2 (40.0)		
Steroid use^e^				
No	100	50 (50.0)	0.415	1
Yes	875	475 (54.3)		1.187 (0.785, 1.796)
Unknown	6	2 (33.3)		–
History of rituximab treatment				
No	735	411 (55.9)	0.023	1
Yes	244	116 (47.5)		0.714 (0.534, 0.955)
Unknown	2	0 (0.0)		–

*ADR* adverse drug reaction, *OR* odds ratio

^a^Chi squared test

^b^Subgroups with a small number of patients were not included in the statistical analysis

^c^If rituximab was administered at ≥4 weeks from the previous administration, it was considered a separate treatment

^d^First dose

^e^Within 24 weeks of the start of rituximab treatment

Among the 981 patients included in the safety analysis set, 319 (32.5%) patients experienced infection/infestation events. Of those, 235 (24.0%) patients experienced ADRs of infections/infestation that were considered related to rituximab treatment. ADRs of Grade ≥ 3 infection/infestation were reported for 48 (4.9%) patients, those of Grade 4 were reported in two (0.2%) patients, and one of Grade 5 was reported in a single patient (0.1%) (Table 3). Specific pathogens causing ADRs of infections and infestations are shown in Online Resource 4. Most infections and infestations were caused by unknown pathogens, and among the known pathogens, influenza was most common. ADRs of infections and infestations are shown according to risk factor for infection in Online Resource 5.

**Table 3:** Incidence of adverse drug reactions of infections/infestations considered related to rituximab treatment (safety analysis set, *N* = 981)

	Infections/infestations *n* (%)	No. ADR events
All grades	235 (24.0)	359
Grade ≥ 3	48 (4.9)	62
Grade 1	105 (10.7)	162
Grade 2	69 (7.0)	120
Grade 3	45 (4.6)	59
Grade 4	2 (0.2)	2
Grade 5	1 (0.1)	1
Grade unknown	13 (1.3)	15

*ADR* adverse drug reaction

Infusion reactions were observed in 313 (31.9%) patients. Ten serious infusion reaction events were observed in four patients (dyspnea, 2; wheezing, 1; hypoxia, 1; oropharyngeal pain, 1; erythema, 1; blood pressure increased, 1; headache, 1; abdominal pain, 1; tachycardia, 1). All patients recovered from all events. The frequency of infusion reactions decreased as the number of rituximab doses increased (Online Resource 6). The incidence of infusion reactions according to infusion conditions during the initial administration of rituximab is shown in Online Resource 7 and infusion reactions according to patient population characteristics are shown in Online Resource 8.

##### Relapse-free period

**Fig. 2:**
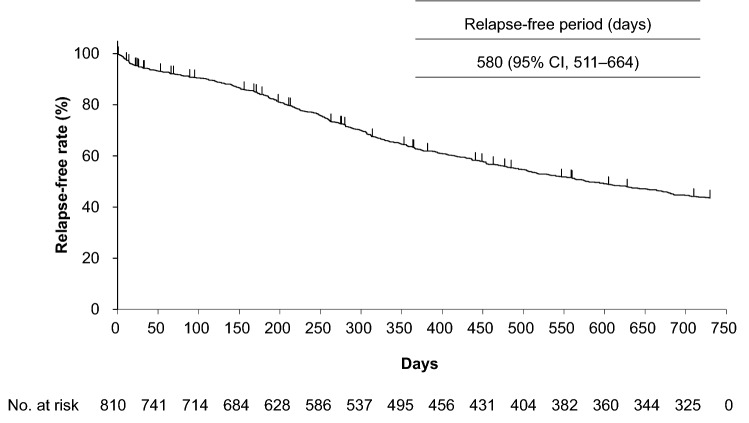
Kaplan–Meier plot of the relapse-free period. *CI* confidence interval

The relapse-free period was 580 days (95% CI, 511–664; Fig. 2). The 50% relapse-free period according to patient background factors is shown in Online Resource 9. Patients who received a single dose of rituximab had a shorter 50% relapse-free period (312 days [95% CI, 285–375]) compared with those who received two doses (479 days [95% CI, 406–627]).

#### Secondary outcome

##### Degree of growth

**Fig. 3:**
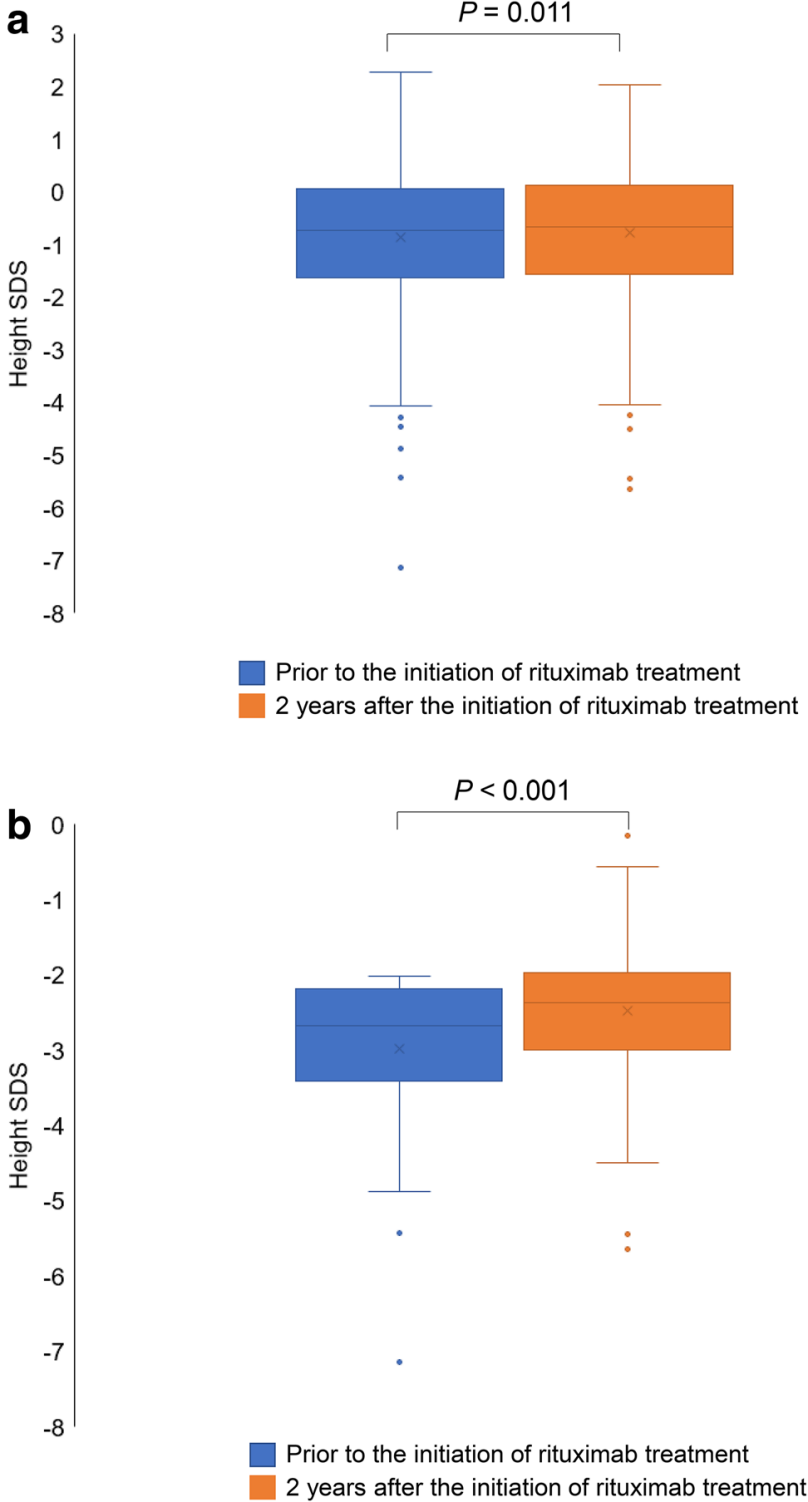
Change in height prior to the initiation of rituximab treatment vs 2 years after the initiation of rituximab treatment in the **a** total pediatric patient population (*n* = 321) and **b** pediatric patients considered to be of low height (SDS < − 2.0) prior to rituximab treatment (*n* = 61). × indicates the mean; the *horizontal line* within each colored box (*blue or orange*) indicates the median *SDS* standard deviation score

Among the pediatric patients for whom height data were available both prior to initiating rituximab treatment and at 2 years after initiating treatment (*n* = 321, excluding patients who were under off-label use), there was a significant change in height SDS (mean change, 0.091; standard deviation [SD], 0.640; *P* = 0.011; Fig. 3a) indicating an improvement over time. Among pediatric patients who were considered to be of low height (SDS < − 2.0) prior to rituximab treatment (*n* = 61), there was a significant change in height SDS (mean change, 0.508; SD, 0.966, *P* < 0.001; Fig. 3b).

## Discussion

Our post-marketing surveillance study of the real-world safety and efficacy of rituximab in patients with refractory nephrotic syndrome found that the incidence and severity of ADRs of infections and infestations was manageable, with Grade ≥ 3 ADRs of infections and infestations being reported in 4.9% of patients over the 2-year study period. We report a relapse-free period of 580 days (all patients) and a significant improvement in the height SDS score in pediatric patients.

The ADRs reported in this study were in line with the known safety profile of rituximab [9]. No safety concerns unique to the use of rituximab in patients with refractory nephrotic syndrome were identified in this analysis. The incidence and severity of ADRs of infection/infestation were lower in our study (24.0%, 235 of 981 patients; Grade ≥ 3: 4.9%, 48 of 981 patients) compared with that reported for previous clinical trials (90.7%, 49 of 54 patients; Grade ≥ 3: 9.3%; 5 of 54 patients; data calculated using the results of RCRNS-01 and RCRNS-02). In the present study, infusion reactions (which were defined as events for which a relationship with rituximab could not be denied) occurred in 313 of the 981 patients included in the safety analysis population (31.9%). In pediatric patients, the incidences were 38.8% (38 of 98 patients) and 41.8% (145 of 347 patients) in the < 7-year old and ≥ 7- to < 15-year old age groups, respectively, which was higher than that observed for adult patients (24.3%). However, the incidence rate in pediatric patients was much lower than the 71.0% (22 of 31) reported in the RCRNS-01 study, which included patients aged 2 years or older. The precise reason for this difference between studies is unclear. Regardless, we think that there are no significant issues related to infusion reactions.

Patient characteristics significantly associated with the incidence of ADRs included initial rituximab dose (375 mg/m^2^), a higher number of rituximab administrations, < 18 years of age at the onset of refractory nephrotic syndrome, no prior rituximab treatment, and concomitant immunosuppressant drugs. A possible explanation for the higher incidence of ADRs in patients who had an initial rituximab dose of 375 mg/m^2^, compared with those treated with 500 mg/person, is that the dose after body surface area adjustment was ≤ 375 mg/m^2^ for most of the patients treated with 500 mg/person (504 of 566; median: 325.9 mg/m^2^; excludes eight patients for whom body surface area could not be calculated). The occurrence of infusion reactions tended to increase with dose per body surface area, which is typical for rituximab. The incidence of ADRs (Table 2), but not the number of serious ADRs (Online Resource 3), was weakly correlated with the number of rituximab administrations. Pediatric patients have lower serum immunoglobulin levels compared with adults, which results in an inability to obtain lifelong immunity and makes them more susceptible to infection [14]. This increased susceptibility to infection (which was considered an ADR) likely contributed to the association of a higher incidence of ADRs in patients who were <18 years of age at the onset of refractory nephrotic syndrome. Increased susceptibility to infection also likely explains the association of concomitant immunosuppressive drugs and a higher incidence of ADRs. Finally, selection bias is likely responsible for the association of no prior rituximab treatment and an increase in ADRs, as patients who had an ADR to rituximab are less likely to get additional treatment.

Of particular note, we report a longer relapse-free period (580 days; 95% CI, 511–664; 810 patients) compared with the 267 days (95% CI, 223–374; 24 patients) reported for a previous clinical study [10], indicating that real-world rituximab use may result in better outcomes than previously thought. The larger size of the present study, the status of underlying disease before treatment, the impact of concomitant treatment with immunosuppressive drugs, and the nature of a real-world vs randomized controlled clinical study likely contributed to this difference. Additionally, rituximab was used after relapse in the clinical study, while in our real-world study, rituximab was used in patients for whom it was deemed necessary by the treating physician, regardless of relapse. An international, multicenter, retrospective cohort study conducted by Chan et al. [15], in children with steroid-dependent/frequent relapsing nephrotic syndrome who were treated with a combination of rituximab (375 mg/m^2^) and maintenance immunosuppression, reported a prolongation of the relapse-free period of 14 months and observed that adverse events were generally mild. Our findings were similar to those of Chan et al., thus confirming the safety and efficacy of rituximab plus maintenance immunosuppression for the treatment of steroid-dependent/frequent relapsing nephrotic syndrome in Japanese pediatric patients. Katsuno et al. reported that rituximab was safe and effective in a study of eight patients with adult-onset SDNS [16]; however, the present study did not aim to determine efficacy in this patient population.

Patients with refractory nephrotic syndrome are often treated with a prolonged course of steroid therapy, which frequently results in growth retardation [17]. It has been reported that the use of rituximab in pediatric patients with SDNS may contribute to improved growth in some patients, as evidenced by a significant change in height SDS after rituximab treatment *(P* = 0.03; height SDS before vs after rituximab treatment; *N* = 13) [17]. We have confirmed this finding in our larger, real-world study (*P* = 0.009; mean change in height SDS before vs after rituximab treatment; *n* = 328).

## Limitations

The analysis in our study was limited in that it only included Japanese patients; the generalizability of these data to other populations and regions remains to be determined. Additionally, as is the nature of post-marketing surveillance studies, the validity of the data included in the analysis was reliant on accurate reporting from the treating physicians.

## Conclusions

The results of the present study demonstrated a lower incidence of ADRs and a longer relapse-free period compared with the previous clinical study of rituximab for the treatment of refractory nephrotic syndrome in Japanese patients. The safety profile of rituximab in this patient population was similar to the known rituximab safety profile and no new safety concerns were identified. These results confirm the safety and efficacy of rituximab treatment in Japanese patients with refractory nephrotic syndrome, both among pediatric and adult patients, and support the continued use of rituximab for their treatment.

## Supplementary Information

Below is the link to the electronic supplementary material.

Supplementary file1 (DOCX 58 KB)
