# Supplementary material for: All-case Japanese post-marketing surveillance of the real-world safety and efficacy of rituximab treatment in patients with refractory nephrotic syndrome
Source: Clin Exp Nephrol. 2021 Apr 1;25(8):854–64. doi: 10.1007/s10157-021-02035-6 (PMC8260412; doi:10.1007/s10157-021-02035-6)
Supplement: Supplementary file 1 — Supplementary file1 (DOCX 58 KB) [file 10157_2021_2035_MOESM1_ESM.docx]

**All-case Japanese post-marketing surveillance of the real-world safety and efficacy of rituximab treatment in patients with refractory nephrotic syndrome**

*Clinical and Experimental Nephrology*

Mana Kobayashi, Yutaro Kageyama, Takashi Ando, Junko Sakamoto, Shohji Kimura

**Corresponding author:**

Takashi Ando

Pharmacovigilance & Quality Assurance Department, Zenyaku Kogyo Co., Ltd., Tokyo, Japan

Email: Takashi_Ando@mail.zenyaku.co.jp

**Online Resources**

**Online Resource 1** Analysis set exclusion criteria

**Safety analysis exclusion criteria:**

• Patients who did not receive any doses of rituximab after registration

• Patients with an unrecoverable questionnaire

• Cases collected outside of the survey period

• Cases for which adverse event data were missing or unknown

• Cases with an invalid investigator signature

• Cases judged to be inappropriate for safety evaluation

**Efficacy analysis exclusion criteria:**

• Data regarding relapse date and/or number of relapses were not available

• Cases in which rituximab was administered for an off-label indication

• Cases judged to be inappropriate for efficacy evaluation

**Online Resource 2** Major reported complications and medical history of the patient population

|  | **Patients**  ***N*** | **Pediatric**  ***n*** | **Adult**  ***n*** |
| --- | --- | --- | --- |
| **Major reported complications** | | | |
| Vascular disorders |  |  |  |
| Hypertension | 256 | 108 | 148 |
| Musculoskeletal and connective tissue disorders |  |  |  |
| Osteoporosis | 151 | 66 | 85 |
| Metabolism and nutrition disorders |  |  |  |
| Dyslipidemia | 65 | 3 | 62 |
| Hyperlipidemia | 79 | 16 | 63 |
| Obesity | 36 | 26 | 10 |
| Steroid diabetes | 29 | 4 | 25 |
| Diabetes mellitus | 17 | 0 | 17 |
| Hypercholesterolemia | 15 | 2 | 13 |
| Blood uric acid increased | 86 | 6 | 80 |
| Eye disorders |  |  |  |
| Cataract | 63 | 28 | 35 |
| Glaucoma | 42 | 30 | 12 |
| Respiratory, thoracic and mediastinal disorders |  |  |  |
| Asthma | 32 | 26 | 6 |
| Rhinitis allergic | 46 | 26 | 20 |
| Psychiatric disorders |  |  |  |
| Insomnia | 27 | 0 | 27 |
| Skin and subcutaneous tissue disorders |  |  |  |
| Dermatitis atopic | 25 | 11 | 14 |
| Gastrointestinal disorders |  |  |  |
| Constipation | 21 | 7 | 14 |
| Immune system disorders |  |  |  |
| Hypogammaglobulinemia | 20 | 10 | 10 |
| Blood and lymphatic system disorders |  |  |  |
| Iron deficiency anemia | 15 | 2 | 13 |
| Clinical examination |  |  |  |
| Body height below normal | 35 | 23 | 12 |
| **Medical history** |  |  |  |
| Respiratory, thoracic and mediastinal disorders |  |  |  |
| Asthma | 30 | 18 | 12 |
| Eye disorders |  |  |  |
| Cataract | 28 | 1 | 27 |
| Glaucoma | 16 | 9 | 7 |
| Vascular disorders |  |  |  |
| Hypertension | 27 | 13 | 14 |
| Injury, poisoning and procedural complications |  |  |  |
| Spine compression facture | 16 | 3 | 13 |
| Infections and infestations |  |  |  |
| Appendicitis | 12 | 0 | 12 |
| Musculoskeletal and connective tissue disorders |  |  |  |
| Osteoporosis | 10 | 5 | 5 |
| Osteonecrosis | 10 | 1 | 9 |

**Online Resource 3** Occurrence of serious adverse drug reactions according to patient and treatment characteristics (Safety analysis set, *N* = 981)

|  | **Total number of patients**  ***N*** | **Patients experiencing ≥ 1 serious ADR**  ***n* (%)** | ***P* value^a,b^** | **OR (95% CI)^b^** |
| --- | --- | --- | --- | --- |
| Total population | 981 | 106 (10.8) |  |  |
| Sex |  |  |  |  |
| Male | 641 | 60 (9.4) | 0.045 | 1 |
| Female | 340 | 46 (13.5) |  | 1.515 (1.007, 2.280) |
| Age (years) |  |  |  |  |
| < 7 | 98 | 30 (30.6) | < 0.001 | 1 |
| 7 to < 15 | 347 | 38 (11.0) |  | 0.279 (0.161, 0.481) |
| ≥ 15 | 536 | 38 (7.1) |  | 0.173 (0.101, 0.297) |
| No. rituximab treatments^c^ |  |  |  |  |
| 1 | 315 | 29 (9.2) | 0.402 | 1 |
| 2 | 232 | 24 (10.3) |  | 1.138 (0.644, 2.011) |
| 3 | 166 | 21 (12.7) |  | 1.428 (0.787, 2.592) |
| 4 | 187 | 17 (9.1) |  | 0.986 (0.526, 1.848) |
| 5 | 61 | 10 (16.4) |  | 1.934 (0.888, 4.210) |
| 6 | 15 | 4 (26.7) |  | - |
| 7 | 3 | 1 (33.3) |  | - |
| 8 | 1 | 0 (0.0) |  | - |
| 9 | 1 | 0 (0.0) |  | - |
| Dose of rituximab (first use) |  |  |  |  |
| 375 mg/m^2^ | 255 | 41 (16.1) | <0.001 | 1 |
| 500 mg/person | 574 | 40 (7.0) |  | 0.391 (0.246, 0.622) |
| 450 mg/person | 10 | 0 (0.0) |  | - |
| 300 mg/person | 19 | 6 (31.6) |  | - |
| 200 mg/person | 15 | 3 (20.0) |  | - |
| 100 mg/person | 43 | 7 (16.3) |  | - |
| Other | 65 | 9 (13.9) |  | - |
| No. rituximab administrations |  |  |  |  |
| 1 | 183 | 16 (8.7) | 0.735 | 1 |
| 2 | 167 | 18 (10.8) |  | 1.261 (0.621, 2.561) |
| 3 | 113 | 11 (9.7) |  | 1.126 (0.503, 2.521) |
| 4 | 287 | 29 (10.1) |  | 1.173 (0.618, 2.226) |
| 5 | 100 | 16 (16.0) |  | 1.988 (0.948, 4.171) |
| 6 | 53 | 6 (11.3) |  | 1.332 (0.494, 3.595) |
| 7 | 29 | 4 (13.8) |  | 1.670 (0.516, 5.400) |
| 8 | 35 | 3 (8.6) |  | 0.979 (0.269, 3.554) |
| 9 | 6 | 2 (33.3) |  | - |
| 10 | 2 | 0 (0.0) |  | - |
| 11 | 2 | 0 (0.0) |  | - |
| 12 | 3 | 0 (0.0) |  | - |
| 16 | 1 | 1 (100.0) |  | - |
| Initial infusion rate^d^ (mg/h) |  |  |  |  |
| 25 | 774 | 78 (10.1) | 0.645 | 1 |
| 50 | 104 | 12 (11.5) |  | 1.164 (0.610, 2.219) |
| < 25 | 63 | 11 (17.5) |  |  |
| 25–50 | 17 | 0 (0.0) |  | - |
| 50–100 | 2 | 1 (50.0) |  | - |
| 100 | 20 | 4 (20.0) |  | - |
| Unknown | 1 | 0 (0.0) |  | - |
| Max infusion rate^d^ (mg/h) |  |  |  |  |
| 200 | 608 | 60 (9.9) | 0.727 | 1 |
| 400 | 25 | 3 (12.0) |  | 1.245 (0.362, 4.284) |
| < 100 | 98 | 15 (15.3) |  | - |
| 100 | 194 | 23 (11.9) |  | - |
| 100–200 | 41 | 4 (9.8) |  | - |
| 200–400 | 13 | 0 (0.0) |  | - |
| Unknown | 2 | 1 (50.0) |  | - |
| Age at refractory nephrotic syndrome onset (years) |  |  |  |  |
| < 18 | 834 | 90 (10.8) | 0.852 | 1 |
| ≥ 18 | 146 | 15 (10.3) |  | 0.947 (0.531, 1.686) |
| Congenital | 1 | 1 (100.0) |  | - |
| Immunosuppressant use^e^ |  |  |  |  |
| No | 106 | 3 (2.8) | 0.006 | 1 |
| Yes | 870 | 101 (11.6) |  | 4.509 (1.404, 14.479) |
| Unknown | 5 | 2 (40.0) |  |  |
| Steroid use^e^ |  |  |  |  |
| No | 100 | 8 (8.0) | 0.362 | 1 |
| Yes | 875 | 96 (11.0) |  | 1.417 (0.667, 3.009) |
| Unknown | 6 | 2 (33.3) |  | - |
| History of rituximab treatment |  |  |  |  |
| No | 735 | 81 (11.0) | 0.739 | 1 |
| Yes | 244 | 25 (10.3) |  | 0.922 (0.574, 1.480) |
| Unknown | 2 | 0 (0.0) |  | - |

*ADR* adverse drug reaction, *OR* odds ratio

^a^Chi-squared test

^b^Subgroups with a small number of patients were not included in the statistical analysis

^c^If rituximab was administered at ≥ 4 weeks from the previous administration, it was considered a separate treatment

^d^First dose

^e^Within 24 weeks of the start of rituximab treatment

**Online Resource 4** Adverse drug reactions of infections/infestations according to pathogen

|  | **Patients**  ***N* (%)** | **Events**  ***n*** |
| --- | --- | --- |
| Influenza | 40 (4.1) | 45 |
| Varicella | 14 (1.4) | 16 |
| Herpes simplex | 10 (1.0) | 12 |
| Streptococcus | 8 (0.8) | 11 |
| Mycoplasma | 7 (0.7) | 7 |
| Pneumococcus | 2 (0.2) | 5 |
| Cytomegalovirus | 5 (0.5) | 5 |
| Norovirus | 4 (0.4) | 4 |
| Candida | 4 (0.4) | 4 |
| *Escherichia coli* | 2 (0.2) | 3 |
| Other | 19 (1.9) | 19 |
| Unknown | 176 (17.9) | 312 |

**Online Resource 5** Incidence of adverse drug reaction of infections/infestations according to risk factor

|  | **Patients**  ***N*** | **ADR of infections/infestations**  ***n* (%)** | **Patients**  ***N^a^*** | **ADR of infections/infestations Grade ≥ 3**  ***n^a^* (%)** |
| --- | --- | --- | --- | --- |
| All patients | 981 | 235 (24.0) | 968 | 48 (5.0) |
| Age (years) |  |  |  |  |
| < 7 | 98 | 44 (44.9) | 95 | 14 (14.7) |
| 7 to < 15 | 347 | 87 (25.1) | 342 | 18 (5.3) |
| ≥ 15 | 536 | 104 (19.4) | 531 | 16 (3.0) |
| Infant (years) |  |  |  |  |
| < 3 | 5 | 2 (40.0) | 5 | 0 (0.0) |
| ≥ 3 | 976 | 233 (23.9) | 963 | 48 (5.0) |
| Elderly (years) |  |  |  |  |
| < 65 | 964 | 228 (23.7) | 952 | 44 (4.6) |
| ≥ 65 | 17 | 7 (41.2) | 16 | 4 (25.0) |
| Age at nephrotic syndrome onset (years) |  |  |  |  |
| < 18 | 834 | 203 (24.3) | 822 | 39 (4.7) |
| ≥ 18 | 146 | 31 (21.2) | 145 | 8 (5.5) |
| Congenital | 1 | 1 (100.0) | 1 | 1 (100.0) |
| Immunosuppressant use^b^ |  |  |  |  |
| No | 106 | 14 (13.2) | 104 | 1 (1.0) |
| Yes | 870 | 219 (25.2) | 860 | 46 (5.3) |
| Unknown | 5 | 2 (40.0) | 4 | 1 (25.0) |
| Steroid use^b^ |  |  |  |  |
| No | 100 | 22 (22.0) | 100 | 3 (3.0) |
| Yes | 875 | 211 (24.1) | 863 | 44 (5.1) |
| Unknown | 6 | 2 (33.3) | 5 | 1 (20.0) |

*ADR* adverse drug reaction

^a^Patients with an unknown grade of ADR were excluded

^b^Within 24 weeks of the start of rituximab treatment

**Online Resource 6** Incidence of infusion reactions according to number of rituximab administrations

|  | **IR** | | | |
| --- | --- | --- | --- | --- |
|  | ***n* [IR] / *N* [administered rituximab] (%)** | | | |
|  | **No. rituximab administrations** | | | |
| Rituximab treatment | **1** | **2** | **3** | **4** |
| 1^st^ | 267/981 (27.2) | 10/324 (3.1) | 11/227 (4.9) | 3/210 (1.4) |
| 2^nd^ | 89/666 (13.4) | 0/100 (0) | 0/40 (0) | 0/29 (0) |
| 3^rd^ | 49/434 (11.3) | 1/29 (3.5) | 0/12 (0) | 0/12 (0) |
| 4^th^ | 24/268 (9.0) | 0/7 (0) | 0/2 (0) | 0/2 (0) |
| 5^th^ | 4/81 (4.9) | 0/1 (0) | 0 | 0 |

*IR* infusion reaction

**Online Resource 7** Incidence of infusion reactions according to the infusion conditions of the initial administration of rituximab

|  | **Patients**  ***N*** | **Patients with IR onset**  ***n*** | **Incidence of IR**  **(%)** |
| --- | --- | --- | --- |
| All patients | 981 | 267 | 27.2 |
| Initial rituximab dose |  |  |  |
| 375 mg/m^2^ | 255 | 88 | 34.5 |
| 500 mg/person | 574 | 145 | 25.3 |
| Other | 152 | 34 | 22.4 |
| Starting infusion rate (mg/h, at first use) |  |  |  |
| < 25 | 63 | 11 | 17.5 |
| 25 | 774 | 227 | 29.3 |
| 25–50 | 17 | 1 | 5.9 |
| 50 | 104 | 27 | 26.0 |
| 50–100 | 2 | 0 | 0.0 |
| 100 | 20 | 1 | 5.0 |
| Unknown | 1 | 0 | 0.0 |
| Max. infusion rate (mg/h, at first use) |  |  |  |
| < 100 | 98 | 29 | 29.6 |
| 100 | 194 | 88 | 45.4 |
| 100–200 | 41 | 14 | 34.2 |
| 200 | 608 | 133 | 21.9 |
| 200–400 | 13 | 2 | 15.4 |
| 400 | 25 | 1 | 4.0 |
| Unknown | 2 | 0 | 0 |
| Patient received premedication |  |  |  |
| No | 4 | 1 | 25.0 |
| Yes | 975 | 266 | 27.3 |
| Unknown | 2 | 0 | 0 |

*IR* infusion reaction

**Online Resource 8** Incidence of infusion reactions according to patient population characteristics

|  | **Patients**  ***N*** | **Patients with IR onset**  ***n*** | **Incidence of IR**  **(%)** |
| --- | --- | --- | --- |
| All patients | 981 | 313 | 31.9 |
| Age (years) |  |  |  |
| < 15 | 445 | 183 | 41.1 |
| ≥ 15 | 536 | 130 | 24.3 |
| Cardiac dysfunction |  |  |  |
| No | 977 | 313 | 32.0 |
| Yes | 4 | 0 | 0.0 |
| Pulmonary dysfunction |  |  |  |
| No | 981 | 313 | 31.9 |
| Yes | 0 | - | - |
| Drug hypersensitivity |  |  |  |
| No | 930 | 293 | 31.5 |
| Yes | 51 | 20 | 39.2 |
| Treatment with an antihypertensive drug |  |  |  |
| No | 704 | 10 | 1.4 |
| Yes | 277 | 5 | 1.8 |

*IR* infusion reaction

**Online Resource 9** Fifty percent relapse-free period according to patient population characteristics

|  | **Patients**  ***N*** | **Patients who experienced relapse or death**  ***n*** | **50% relapse-free period**  **(estimated days [95% CI])** |
| --- | --- | --- | --- |
| Sex |  |  |  |
| Male | 546 | 300 | 517 (454, 627) |
| Female | 264 | 132 | 666 (572, -) |
| Age at start of administration (years) |  |  |  |
| < 7 | 94 | 60 | 377 (285, 506) |
| 7 to < 15 | 335 | 223 | 406 (341, 498) |
| ≥ 15 | 381 | 149 | - (-, -) |
| No. relapses per year prior to rituximab administration |  |  |  |
| 0–1 | 195 | 73 | - (-, -) |
| 2–3 | 318 | 170 | 605 (490, 681) |
| ≥ 4 | 263 | 170 | 358 (307, 437) |
| No. rituximab treatments prior to relapse^a^ |  |  |  |
| 1 | 549 | 377 | 358 (321, 412) |
| 2 | 90 | 43 | 685 (581, -) |
| ≥ 3 | 168 | 11 | - (-, -) |
| Dose of rituximab (first use) |  |  |  |
| 375 mg/m^2^ | 234 | 136 | 506 (419, 628) |
| 500 mg/person | 439 | 199 | - (664, -) |
| No. doses of rituximab prior to relapse |  |  |  |
| 1 | 322 | 219 | 312 (285, 375) |
| 2 | 132 | 82 | 479 (406, 627) |
| 3 | 51 | 14 | - (-, -) |
| 4 | 252 | 114 | - (603, -) |
| ≥ 5 | 50 | 2 | - (-, -) |
| Initial infusion rate^b^ |  |  |  |
| 50 mg/h | 71 | 36 | 599 (367, -) |
| 25 mg/h | 642 | 336 | 619 (540, 686) |
| Reason for rituximab use |  |  |  |
| Frequent relapse | 246 | 140 | 484 (395, 619) |
| Steroid dependence | 311 | 166 | 599 (514, 685) |
| Frequent relapse/steroid dependence | 253 | 126 | 672 (507, -) |
| Disease type |  |  |  |
| Minimal change disease | 263 | 125 | 699 (575, -) |
| Focal segmental glomerulosclerosis | 37 | 9 | - (-, -) |
| Membranous nephropathy | 1 | 0 | - (-, -) |
| Membranoproliferative glomerulonephritis | 1 | 0 | - (-, -) |
| Other | 5 | 2 | - (563, -) |
| Previous treatment |  |  |  |
| No | 1 | 0 | - (-, -) |
| Yes | 809 | 432 | 577 (511, 664) |
| History of rituximab treatment |  |  |  |
| No | 598 | 332 | 544 (472, 619) |
| Yes | 211 | 99 | 724 (564, -) |
| Immunosuppressant use^c^ |  |  |  |
| No | 67 | 23 | - (-, -) |
| Yes | 742 | 408 | 560 (490, 630) |
| Steroid use^c^ |  |  |  |
| No | 81 | 32 | - (605, -) |
| Yes | 727 | 399 | 554 (488, 630) |
| Cardiac dysfunction |  |  |  |
| No | 809 | 432 | 577 (511, 664) |
| Yes | 1 | 0 | - (-, -) |
| Drug hypersensitivity |  |  |  |
| No | 769 | 409 | 581 (516, 670) |
| Yes | 41 | 23 | 428 (214, -) |
| Treatment with an antihypertensive drug |  |  |  |
| No | 596 | 321 | 564 (494, 664) |
| Yes | 214 | 111 | 643 (504, -) |
| Vaccine use |  |  |  |
| No | 808 | 431 | 580 (511, 664) |
| Yes | 2 | 1 | - (252, -) |

*CI* confidence interval

^a^If rituximab was administered at ≥ 4 weeks from the previous administration, it was considered a separate treatment

^b^First dose

^c^Within 24 weeks of the start of rituximab treatment
